# Short-Term Plasticity in Cortical GABAergic Synapses on Olfactory Bulb Granule Cells Is Modulated by Endocannabinoids

**DOI:** 10.3389/fncel.2021.629052

**Published:** 2021-02-09

**Authors:** Fu-Wen Zhou, Adam C. Puche

**Affiliations:** Department of Anatomy and Neurobiology, Program in Neurosciences, University of Maryland School of Medicine, Baltimore, MD, United States

**Keywords:** Endocannabinoids, olfactory bulb, short term plasticity, GABA, diagonal band of Broca, olfactory cortex, feedback

## Abstract

Olfactory bulb and higher processing areas are synaptically interconnected, providing rapid regulation of olfactory bulb circuit dynamics and sensory processing. Short-term plasticity changes at any of these synapses could modulate sensory processing and potentially short-term sensory memory. A key olfactory bulb circuit for mediating cortical feedback modulation is granule cells, which are targeted by multiple cortical regions including both glutamatergic excitatory inputs and GABAergic inhibitory inputs. There is robust endocannabinoid modulation of excitatory inputs to granule cells and here we explored whether there was also endocannabinoid modulation of the inhibitory cortical inputs to granule cells. We expressed light-gated cation channel channelrhodopsin-2 (ChR2) in GABAergic neurons in the horizontal limb of the diagonal band of Broca (HDB) and their projections to granule cells in olfactory bulb. Selective optical activation of ChR2 positive axons/terminals generated strong, frequency-dependent short-term depression of GABA_*A*_-mediated-IPSC in granule cells. As cannabinoid type 1 (CB1) receptor is heavily expressed in olfactory bulb granule cell layer (GCL) and there is endogenous endocannabinoid release in GCL, we investigated whether activation of CB1 receptor modulated the HDB IPSC and short-term depression at the HDB→granule cell synapse. Activation of the CB1 receptor by the exogenous agonist Win 55,212-2 significantly decreased the peak amplitude of individual IPSC and decreased short-term depression, while blockade of the CB1 receptor by AM 251 slightly increased individual IPSCs and increased short-term depression. Thus, we conclude that there is tonic endocannabinoid activation of the GABAergic projections of the HDB to granule cells, similar to the modulation observed with glutamatergic projections to granule cells. Modulation of inhibitory synaptic currents and frequency-dependent short-term depression could regulate the precise balance of cortical feedback excitation and inhibition of granule cells leading to changes in granule cell mediated inhibition of olfactory bulb output to higher processing areas.

## Introduction

Reciprocal projections between olfactory bulb and brain higher processing areas form a feedback/feedforward circuit that can rapidly regulate the olfactory bulb circuit dynamics and sensory processing (Rothermel et al., 2014; Soria-Gómez et al., 2014; Mazo et al., 2016; In ’t Zandt et al., 2019). These higher processing areas include primary olfactory cortex (piriform cortex, anterior olfactory nucleus, tenia tecta, olfactory tubercle, and entorhinal cortex – all glutamatergic), hippocampal structures (glutamatergic), locus coeruleus (noradrenergic), raphe nucleus (serotonergic), and basal forebrain (cholinergic and GABAergic neurons) (Luskin and Price, 1983; Záborszky et al., 1986; Shipley and Ennis, 1996; Nunez-Parra et al., 2013; Ennis et al., 2015; Sanz Diez et al., 2019). While considerable work has been done on glutamatergic and neuromodulatory inputs to the olfactory bulb, comparatively less is known of the GABAergic cortical inputs.

The diagonal band of Broca, one of the basal forebrain structures, comprises a vertical limb (VDB) and the horizontal limb of the diagonal band of Broca (HDB). Cholinergic and GABAergic neurons are intermingled in HDB (Brashear et al., 1986; Záborszky et al., 1986; Yang et al., 2014) and the density of cholinergic neurons is lower than that of GABAergic neurons (Yang et al., 2014). Cholinergic neurons (Linster and Hasselmo, 2000; Rothermel et al., 2014; Case et al., 2017) and GABAergic neurons (Ma and Luo, 2012; Nunez-Parra et al., 2013; Sanz Diez et al., 2019) send projections to olfactory bulb. Electrical stimulation of olfactory bulb output neuronal axons modulates HDB neuron firing (Linster and Hasselmo, 2000), suggesting HDB and olfactory structures could form a reciprocally interconnected loop. Projections of HDB neurons to olfactory bulb have been shown to influence olfactory processing (Paolini and McKenzie, 1993; Roman et al., 1993; Linster and Hasselmo, 2000; Nunez-Parra et al., 2013; Devore et al., 2016), but the underlying circuit mechanisms are unclear.

Granule cell activation strongly depends on centrifugal input and granule cells may be critically involved in mediating top-down modulation of sensory processing in the main olfactory bulb (Burton, 2017). Granule cells are also the main synaptic targets of centrifugal feedback projections from olfactory cortex (Davis and Macrides, 1981; Luskin and Price, 1983; Carson, 1984; Shipley and Adamek, 1984; Balu et al., 2007; Boyd et al., 2012; Zhou et al., 2018) and receive projections from anterior olfactory nucleus/cortex (Markopoulos et al., 2012). In addition to excitatory input to granule cells, there is also inhibitory centrifugal projections with the HDB sending inhibitory GABAergic projections to granule cells (Ma and Luo, 2012; Nunez-Parra et al., 2013), and a disruption of inhibition impairs olfactory discrimination (Nunez-Parra et al., 2013). Optical activation of HDB GABAergic inhibitory fibers evokes IPSCs in granule cells (Ma and Luo, 2012; Nunez-Parra et al., 2013; Sanz Diez et al., 2019) and other cell types in olfactory bulb including deep short axon cells and periglomerular interneurons (Sanz Diez et al., 2019).

Cannabinoid type 1 (CB1) receptors are expressed at axon terminals of many neuron types throughout the brain and are involved in modulating a wide range of physiologic properties including synaptic transmission, synaptic plasticity, learning and memory, and sensory processing (Marsicano and Lutz, 1999; Soria-Gómez et al., 2014; Araque et al., 2017; Harvey and Heinbockel, 2018). In the olfactory system, endocannabinoids can be released by external tufted cells (Heinbockel et al., 2016), deep short axon cells, granule cells (Pouille and Schoppa, 2018), and mitral cells (Heinbockel and Wang, 2015; Freundt-Revilla et al., 2017). These endocannabinoids can regulate neuronal activity and signaling in olfactory glomerular cells (Wang et al., 2012, 2019; Heinbockel et al., 2016; Pouille and Schoppa, 2018). CB1 receptors are intensely expressed in the network of fibers throughout the granule cell layer (GCL) and a population of cells within the internal GCL (Freundt-Revilla et al., 2017; Wang et al., 2019). Cortical centrifugal glutamatergic terminals have been shown to highly express CB1 receptors in the GCL (Soria-Gómez et al., 2014). Additionally, endocannabinoid regulates excitatory corticofugal input to deep short axon cells and granule cells (Pouille and Schoppa, 2018). However, it remains unknown if HDB GABAergic inhibitory fibers are modulated by CB1 receptor activity.

Here we expressed light-gated cation channel channelrhodopsin-2 (ChR2) in HDB GABAergic neurons and their axons to investigate inhibitory postsynaptic currents/potentials (IPSCs/IPSPs) and short-term plasticity (STP) of the HDB→granule cell synapse, and their modulation by CB1 receptor activation. Selective activation of this synapse generates strong, frequency-dependent paired-pulse depression of IPSP and GABA_*A*_-mediated-IPSC. Activation of CB1 receptor greatly decreased IPSCs and decreased short-term synaptic depression and blockade of tonic endogenous activation of CB1 receptor increased IPSC and increased short-term synaptic depression.

## Materials and Methods

### Ethics Statement

Colony maintenance, animal identification, and all experimental procedures (including virus injection, monitoring after surgery, slice cutting, and endpoints) were ethically conducted in accordance with protocols approved by the University of Maryland Institutional Animal Care and Use Committee.

### Animals and Slice Preparation

Transgenic GAD2-Cre mice were obtained from Jackson Laboratory. Mice were housed in a standard 12-h light/dark cycle with *ad libitum* access to food and water. ChR2 was expressed by injection of Cre-inducible adeno-associated virus serotype 9 (AAV2.9, also known simply as AAV9) carrying fusion genes for ChR2 and enhanced yellow (AAV-hSyn-hChR2(H134R)-EYFP). Briefly, the skull of 8–12 week animals was exposed and small holes (∼0.5 mm diameter; coordination: anteroposterior, AP, + 0.85, mediolateral, ML ± 0.5, dorsoventral, DV, −4.6; AP, + 0.65, ML, ± 0.7, DV, −4.9; AP, + 0.6, ML, ± 0.75, DV, −4.9 mm. Franklin and Paxinos, 2008) drilled for injections in the HDB.

After at least 3 week for ChR2 expression in olfactory bulb, horizontal olfactory bulb slices were cut as previous described (Zhou et al., 2016). Briefly, the brain was quickly dissected from the skull and 350-μm slices cut with a VT1200S Vibratome (Leica Biosystems Nussloch GmbH, Nussloch, Germany) in 4°C oxygenated (95% O_2_–5% CO_2_) cutting solution containing (in mM) 204.5 sucrose, 3 KCl, 1.25 NaH_2_PO_4_, 25 NaHCO_3_, 2.6 MgSO_4_, 0.5 CaCl_2_, and 10 D-glucose. The slices were then transferred to a holding chamber with oxygenated artificial cerebrospinal fluid (ACSF, in mM): 125 NaCl, 3 KCl, 1.25 NaH_2_PO_4_, 25 NaHCO_3_, 1.3 MgSO_4_, 1.3 CaCl_2_, and 10 D-glucose for at least 1-h incubation at 23°C before recording.

### Recording

Slices were transferred to the recording chamber and perfused at 3 ml/min with ACSF. Recordings were made at 30°C (Bipolar Temperature Controller, Norfolk, VA, United States) under visual guidance on a BX50WI (Olympus Corporation, Tokyo, Japan) fixed-stage upright microscope equipped with near-infrared differential interference contrast optics. Granule cells were whole-cell recorded in the superficial GCL (Zhou et al., 2018) as there is emerging understanding that there may be differences in granule cell subpopulations as a function of depth. Recording pipettes were pulled from thick-wall borosilicate glass with filament (O.D. 1.5 mm, I.D. 0.75 mm; Sutter Instrument, Novato, CA, United States) in a horizontal pipette puller (model P-97 Flaming/Brown Micropipette puller; Sutter Instrument, Novato, CA, United States). Patch electrodes had resistances of 7 to 8 MΩ when filled with an internal solution containing (in mM) 125 CsCH_3_SO_3_ for voltage- and 120 K-gluconate for current-clamp recording, with additionally 4 MgCl_2_, 5 EGTA, 10 HEPES, 3 Na_2_-ATP, 0.3 Na_3_-GTP, 4 Na_2_-phosphocreatine and 0.1% biocytin for both (290 mOsm/Kg H_2_O and pH was adjusted to 7.3 with CsOH or KOH). MultiClamp 700A amplifiers, pClamp 9.2 software and Digidata 1322A interface (Molecular Devices, Axon Instruments, San Jose, CA, United States) were used to acquire and analyze data. Signals were digitized at 20 KHz and analyzed offline.

### Optical Stimulation

Green laser flashes were generated by a Diode-Pumped Solid-State Laser (MBL-III-473-100 mW, Opto Engine LLC, UT, United States) coupled to a high speed Uniblitz shutter (Vincent Associates, NY, United States). This shutter generated gated short duration (1.5 ms) optical exposures with stimulations at different frequencies. Laser flashes were delivered through multimode optical fiber (0.1 numerical aperture, 7° beam spread; ThorLabs, Newton, NJ, United States). Optical power delivered at the fiber tip was calibrated with a PM20A Power Meter (ThorLabs, Newton, NJ, United States) and ranged from ∼0.5 to 8.5 mW. The optical stimulation pulses were generated by a PG4000A digital stimulator (Cygnus Technologies, Southport, NC, United States). The different interstimulus’ intervals (or stimulation frequencies) were controlled by a MultiClamp 700A commander. In all five or paired-pulse experiments, different interstimulus intervals were delivered in random order to obviate long-term facilitation/depression-like effects. ChR2 has kinetic difficulties in following frequencies of 40 Hz or greater, at which rate steady-state plateau currents emerge from the ChR2 current of the first stimulation has incompletely returning to baseline by the time the second stimulation occurs. This limited stimulation frequency to 40 Hz or less. The onset and duration of optical stimulation were measured by splitting 1% of the laser beam out to a high speed (30 ns rise time) silicon photosensor (model 818-BB, Newport, Irvine, CA, United States) and were recorded by the same MultiClamp 700A amplifier as the patch electrode. ChR2 expressed centrifugal fibers from HDB GABAergic neurons were activated by green laser optical stimulation and the initiated postsynaptic current or potential recorded at different holding potentials. The amplitude of control pulses across the course of an experiment is monitored to identify if a cell is “running downhill.” Series resistances were between 8 and 15 MΩ and cells were discarded from a further analysis if access resistance changed by >10% with time or on application of drug during the experiment.

### Immunohistology

Following recording, slices with biocytin-filled cells were fixed in 4% paraformaldehyde in 0.1 M phosphate buffer at 4°C overnight. Slices were incubated with 4 μg/mL Alexa Fluor 546 streptavidin for 2 h at room temperature and were then mounted and imaged. Digital microscopy images were captured using a FluoView500 confocal microscope (Olympus Corporation, Tokyo, Japan). Cells were visually classified as being granule cells if they had the typical morphology of short basal dendrites and a long apical dendrite with spines entering the external plexiform layer (Price and Powell, 1970).

### Statistical Analysis

Data were analyzed with Clampfit 9.2 (Molecular Devices, Axon Instruments, San Jose, CA, United States). We calculated the onset synaptic latency of optical stimulation evoked response (postsynaptic current or potential) as time from the onset of light to the onset of the response, and synaptic jitter as the standard deviation of latencies from 20 trials. Peak amplitude was measured as the difference between the baseline current level and the peak of the response; and half-width was defined as the time difference between rising phase and falling phase of the response measured at 50% response peak amplitude. Rise tau was defined as the time taken for the current to rise from 10 to 90% of response peak amplitude; decay tau was the time to decrease from 90 to 10% response peak amplitude. The paired-pulse ratio (PPR) was calculated as the ratio of the response peak amplitude of the test pulse(s) to the control pulse. Statistical analysis, graphs and plotting were completed with Origin 2018 (Originlab Corporation, Northampton, MA, United States). All values were expressed as mean ± SEM. Paired *t*-tests and ANOVA were used to compare data.

### Drugs

All drugs were purchased from Sigma-Aldrich (Cleveland, OH, United States) except that WIN 55,212-2 mesylate (CB1 agonist, 5 μM and others), AM-251 (CB1 antagonist, 5 μM), D-2-amino-5-phosphonovalerate (APV, NMDA receptor antagonist, 50 μM), 1,2,3,4-Tetrahydro-6-nitro-2,3-dioxo-benzo[f]quinoxaline-7-sulfonamide (NBQX, AMPA and kainate receptor antagonist, 10 μM), gabazine (selective GABA_*A*_ receptor antagonist, 10 μM) were purchased from Tocris Cookson (Ellisville, MO, United States). All drugs were bath applied by diluting in ACSF at the above-indicated concentration unless otherwise stated.

## Results

### HDB GABAergic Projections to Olfactory Bulb

**FIGURE 1:**
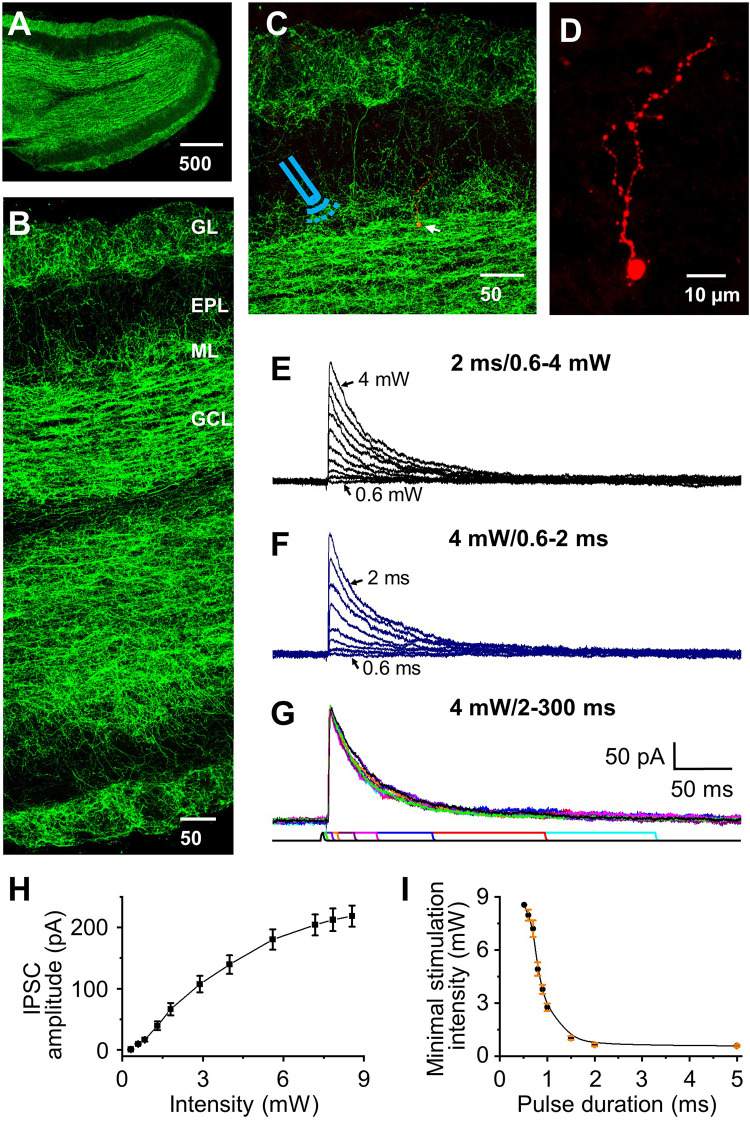
Optical stimulation of HDB-granule cell axons initiates IPSC in granule cells. **(A)** Staining of ChR2 showing distribution of labeled axons throughout the olfactory bulb. **(B)** Higher magnification showing differential distribution of ChR2 labeling in olfactory bulb. From highest density to lowest density: granule cell (GCL), glomerular (GL), mitral cell layer (ML), and external plexiform layer (EPL). **(C)** Double staining of ChR2 (*green*) and biocytin (*red*, **arrow**) filled granule cell, with the ***Icon*** representing the approximate location of the fiber optic on incoming axons to the target cell. **(D)** Higher magnification showing a representative biocytin filled granule cell. **(E)** Representative IPSCs (low to high amplitude) elicited by varying optical stimulation intensities from 0 to 4 mW (0.6, 0.7, 0.8, 1, 1.2, 1.5, 2, 2.5, 3, 4 mW) at a pulse duration of 2 ms. **(F)** Representative traces showing that the IPSC peak amplitude decreases as the pulse duration decreases (2, 1.5, 1.3, 1, 0.9, 0.8, 0.7, and 0.6 ms) stimulated at 4.0 mW. **(G)** Representative traces showing similar IPSC amplitude when the pulse duration was 2 ms or longer (5, 10, 15, 30, 50, 100, 200 and 300 ms) at stimulation intensity of 4.0 mW. **(H)** Group data showing current response curve of different optical stimulation intensities from 0 to 8.5 mW at pulse duration of 2 ms. *N* = 6. **(I)** The minimal intensity at different pulse duration of optical stimulation to initiate a threshold postsynaptic current. The threshold was set as 2 × σ_*noise*_, where σ_*noise*_ was measured during periods of no visually detectable events and was typically 2–4 pA. Optical power delivered power ranges of ∼0.5 to 8.5 mW. *N* = 5.

To label the GABAergic inhibitory projections from the horizontal limb of the diagonal band of Broca (HDB) in the olfactory bulb, we expressed the light-activated channel ChR2 in HDB GABAergic neurons and their projections by injecting AAV-hSyn-hChR2(H134R)-EYFP into the HDB of Gad2-Cre mice. This drove Cre-dependent co-expression of ChR2 and EYFP in GABAergic neurons. ChR2 labeled axons emanating from HDB GABAergic neurons terminated in all layers of olfactory bulb (Figure 1A). The densest labeling was found in GCL and glomerular layer (GL), lower density labeling in mitral cell layer (ML) and only scattered fibers labeling in external plexiform layer (EPL, Figure 1B).

### Optical Stimulation Initiates HDB-Granule Cell Inhibitory Postsynaptic Current

Optical stimulation initiated IPSCs at the holding potential of 0 mV were recorded in olfactory bulbar granule cells, and after recording all cells with biocytin were stained to verify that they matched classic granule cell morphology (Figures 1C,D; Price and Powell, 1970). We varied the optical stimulation intensity from ∼0.5–8.5 mW and any pulse durations ∼0.5 to 300 ms to ensure stimulation power/duration were calibrated to be within the optima for each cell (Figures 1E–G). As expected, IPSC peak amplitude increased with increasing of stimulation intensity (Figures 1E,H) along an asymptoting amplitude to intensity curve (Figure 1H). At durations <2 ms enhanced power was required to elicit IPSCs (Figures 1F,I) and small variations in duration/power exhibiting a significant effect on activation threshold. Thus, we chose to use a pulse duration of 2 ms (used in all subsequent experiments) and calibrated the stimulation intensity used with each cell to obtain IPSC peak amplitude at 70% of the maximal response to examine STP at different frequencies (power range 2–4 mW).

### GABA_*A*_ Mediated HDB-Granule Cell Inhibitory Postsynaptic Current

**FIGURE 2:**
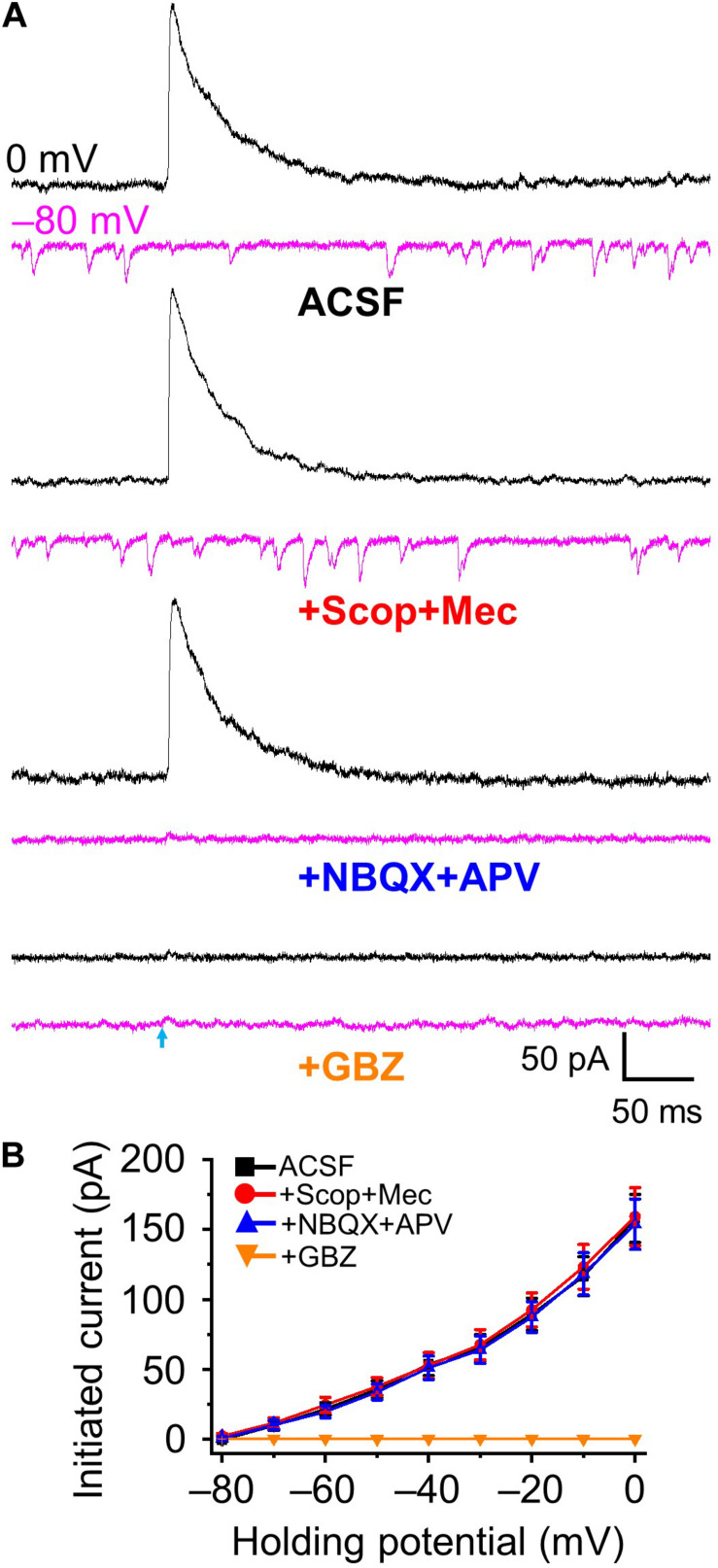
Optical stimulation induced GABA_*A*_-mediated inhibitory postsynaptic currents. **(A)** Example traces showing postsynaptic currents at the holding potential of 0 mV (**black**) and –80 mV (**magenta**) in ACSF, +Scop+Mec (mAChR and nAChR antagonists scopolamine, 10 μM and mecamylamine, 10 μM, respectively), further addition of NBQX+APV (AMPA/kainate receptor antagonist NBQX, 10 μM and NMDA receptor antagonist APV, 50 μM) and further addition of GBZ (gabazine, GABA_*A*_ receptor antagonist, 10 μM). **(B)** Group data showing optical stimulation-initiated currents at holding potentials from 0 to −80 mV in 10 mV increments. *N* = 5.

Gamma aminobutyric acid (GABA) and acetylecholine (ACh) expression from basal forebrain (HDB) acetylcholinergic neurons to olfactory bulb axon terminals has been reported in the literature (Case et al., 2017), and co-release may be a possibility in our recordings. We examined whether optical stimulation of ChR2-expressing inhibitory GABAergic axons/terminals from HDB could cause release of GABA and other neurotransmitter(s). Optical stimulations of GABAergic fibers and recording from granule cells were performed varying the holding potential from 0 mV (enhancing Cl^–^ driving force to maximize inhibitory outward currents) to −80 mV (maximizing inward excitatory currents with minimal Cl^–^ driving force and thus minimize IPSCs) at 10 mV intervals. Stimulation evoked a robust IPSC at 0 mV (157.92 ± 17.21 pA) and negligible outward currents at −80 mV (0.47 ± 1.27 pA, *n* = 5, Figures 2A,B). As expected, IPSCs were completely blocked by GABA_*A*_ receptor antagonist gabazine (10 μM) and spontaneous EPSCs present at −80 mV holding potential completely blocked by NBQX + APV. Although there are literature reports on co-expression of ACh and GABA, the combination of both nAChR (10 μM scopolamine, Scop) and mAChR antagonists (10 μM mecamylamine, Mec) had no significant effect on optically evoked currents at any holding potential from 0 to −80 mV (Figure 2B, all *P* > 0.05, Scop + Mec *vs* ACSF at different holding potentials, respectively). Those results indicate that optically stimulation evokes postsynaptic current IPSCs mediated by GABA_*A*_ receptors, and we observe no evidence of ACh release under these conditions.

### Short-Term Depression of HDB-Granule Cell Inhibitory Postsynaptic Responses

Animals can sniff/respire at low frequency (1 – 3 Hz) in a familiar environment (at rest) or engage in bouts of rhythmic high-frequency sniffing (5–10 Hz) during sampling odors when presented with novel stimuli (Wesson et al., 2009; Wachowiak, 2011). The number of sniffs in a bout is ∼46% possibility of 1 sniff, ∼20% of 2 sniffs, and ∼20% of 5 or more sniffs in mouse (Sirotin et al., 2014). However, in cases only one or two sniffs, depending on the complexity of the discrimination task, is required to elicit behavioral decision responses (Uchida and Mainen, 2003; Mainland and Sobel, 2006). Thus, to recapitulate the majority of sniffs per bout in a behavioral paradigm we examined five stimuli trains at different frequencies to investigate STP of IPSCs at the HDB-granule cell synapse.

**FIGURE 3:**
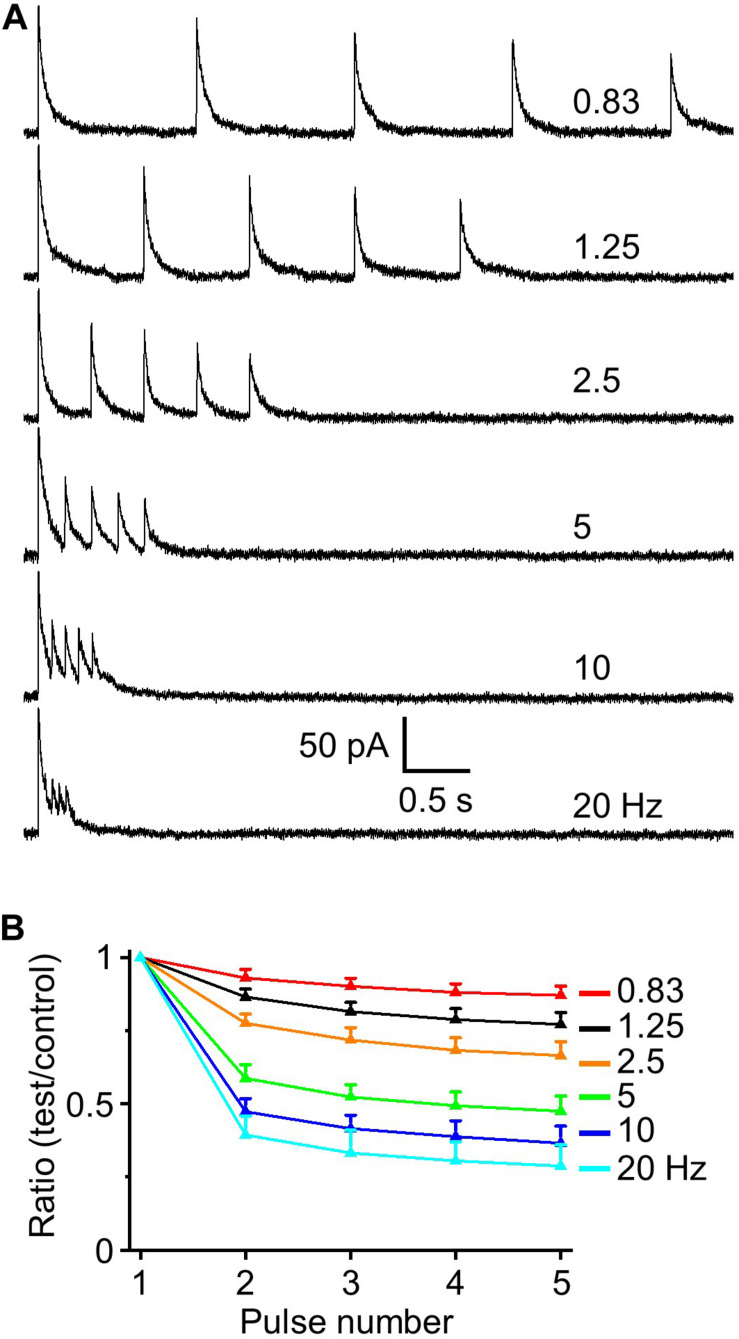
IPSC depression induced by a train of five optical simulations. **(A)** Example traces of evoked IPSCs at interstimulus frequencies from 0.83 to 20 Hz (0.83, 1.25, 2.5, 5, 10, and 20 Hz). **(B)** Population data for five-pulse ratio of IPSC peak amplitude of the test pulses 1–4 to the control first pulse (*n* = 6).

To assess if there is short-term potentiation or depression at HDB-granule cell inhibitory synapse we used five-pulse optical activation of ChR2 labeling axons from HDB at frequencies (0.83 to 20 Hz) spanning over the range of basal respiration (∼1.5 Hz) to maximal sniff rate (∼10 Hz; Wesson et al., 2009; Wachowiak, 2011). STP of IPSCs (recorded at 0 mV unless otherwise stated) was assessed as the ratios of the four-test synaptic peak amplitudes to the control first stimulus. These ratios steadily decreased from 1.0 to smaller with increased stimulation frequencies (Figures 3A,B; e.g., *F*_6_,_35_ = 143.02, *P* < 0.01 for the ratio of test1/control, *n* = 6, one-way ANOVA), indicating short-term depression in IPSC response. Those ratios were significantly depressed when frequencies were 2.5 or higher (*P* < 0.01, paired *t*-test, *n* = 6) with limited effect at frequencies of 0.83 and 1.25 Hz. Interestingly, the greatest depression occurred between the first and second stimulus with little additional short-term depression with stimuli 3–5. This demonstrates that the GABAergic HDB synapse undergoes short-term depression and that this depression is near maximal with just the second stimulus input in a high frequency bout.

**FIGURE 4:**
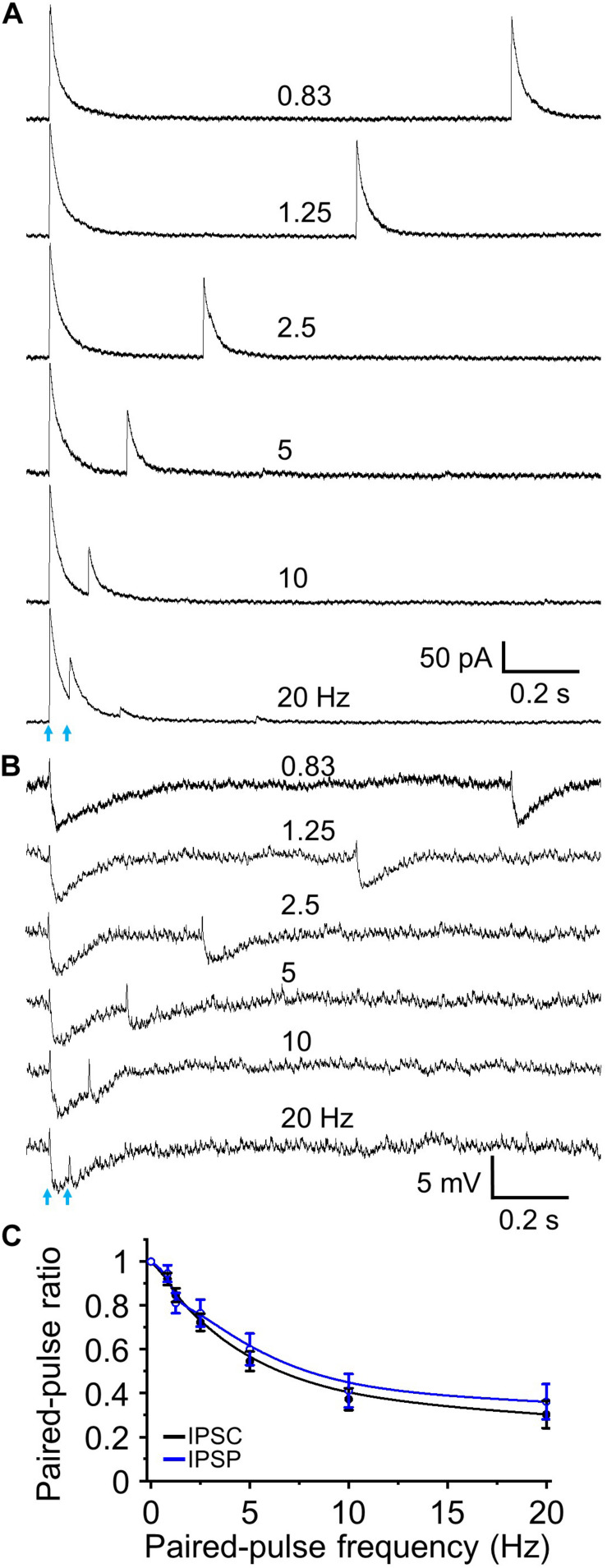
Paired-pulse optical simulation induced short-term synaptic depression of IPSCs and IPSPs. **(A)** Representative traces showing optical stimulation-initiated IPSCs at a holding potential of 0 mV to interstimulus frequencies of 0.83 to 20 Hz. **(B)** Optical stimulation-initiated IPSPs at resting membrane potential at interstimulus frequencies of 0.83 to 20 Hz. The rest membrane potential was –67.3 mV in this example cell. **(C)** Population data for paired pulse ratios of IPSC and IPSP peak amplitude of the test pulse to control. *N* = 9 for IPSCs and *n* = 6 for IPSPs.

Since two stimuli elicited near maximal short-term depression subsequent experiments were performed using a paired-pulse paradigm. This is also a standardized method for evaluating short-term synaptic plasticity in different regions including olfactory bulb (Murphy et al., 2004; Zhou et al., 2020). We obtained PPR (test/control) and observed paired-pulse depression (*n* = 9, Figures 4A,C) that, as expected, had identical magnitude and frequency dependency as the preceding five pulse train experiments. The resting membrane potential of recorded granule cells was −70.76 ± 1.46 mV with an input resistance of 586.3 ± 60.9 MΩ (*n* = 11). At −70 mV optically evoked IPSCs exhibited an outward current of 9.72 ± 3.52 pA (Figure 2B), suggesting this outward current would hyperpolarize the cell by ∼5 mV. Indeed, when we performed current clamp recording and measured IPSPs, optical stimulation induced a hyperpolarization of 5.33 ± 0.47 mV from resting membrane potential (*n* = 6). Performing paired-pulse stimulation recordings from 0.83 to 20 Hz demonstrated a tight concordance between paired-pulse depression of IPSCs and IPSPs (*P* > 0.05 at different frequencies, respectively, one-way ANOVA; Figure 4B,C). These ratios for IPSC and IPSP steadily decreased from 1.0 to smaller with increased stimulation frequencies (*F*_6_,_55_ = 302.51, *n* = 9 for IPSC and *F*_6_,_35_ = 161.16, *n* = 6 for IPSP, *P* < 0.01, one-way ANOVA; Figure 4C).

### Endocannabinoid Receptor Activation Suppresses HDB-Granule Cell Inhibitory Postsynaptic Currents

Endocannabinoids modulate glutamatergic cortical inputs to the olfactory bulb (Wang et al., 2012), but nothing is known on whether they also modulate GABAergic HDB cortical input to the bulb. To examine the role of CB1 receptor activation in the modulation of optically evoked IPSCs, we tested the effect of the CB receptor agonist WIN 55,212-2 on amplitude of IPSCs in granule cells. CB2 is recently reported to express in the brain (Zhang et al., 2014), but not in olfactory bulb (Gong et al., 2006), thus the agonist WIN 55,212-2 is likely acting only at the CB1 receptor in olfactory bulb.

**FIGURE 5:**
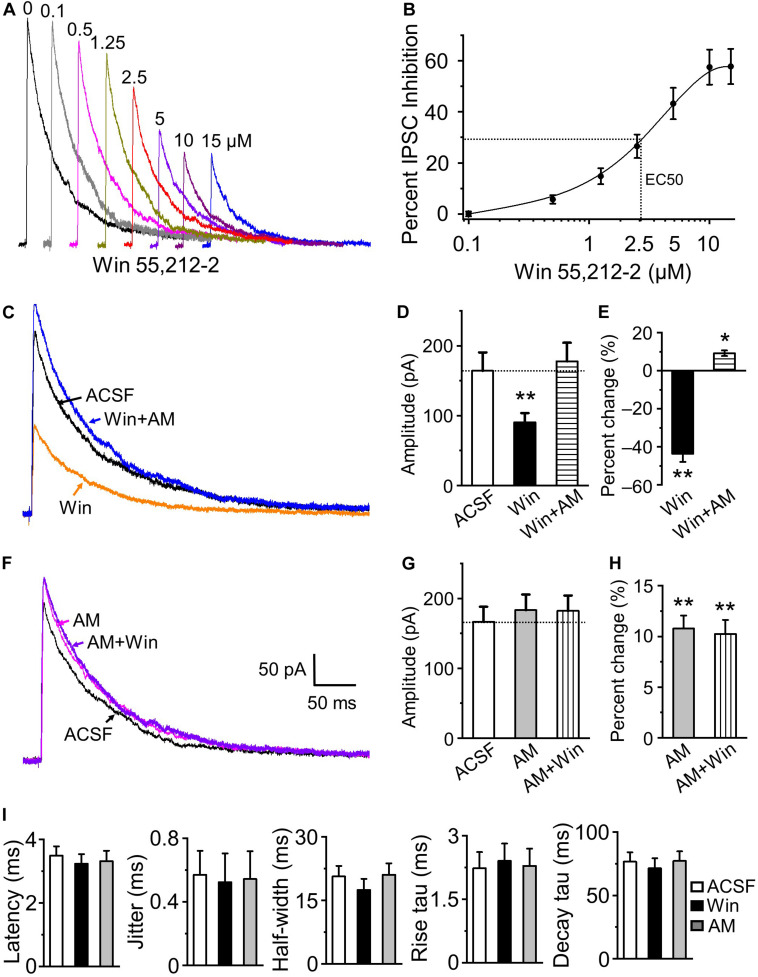
Endocannabinoids modulate the magnitude of granule cell responses to HDB GABAergic input. **(A)** Representative IPSC traces in the presence of bath applied Win 55,212-2 of 0 to 15 μM (0.1, 0.5, 1.25, 2.5, 5, 10, and 15 μM). **(B)** Group data showing dose-response curve of IPSC inhibition by WIN-55,212-2 (*n* = 4 for 0.1 μM to 5 other individual doses) with EC50 at approximately 2.5 μM. **(C)** Representative traces of IPSCs in control ACSF (**black**), the presence of 5 μM Win 55,212-2 (**orange**) and the further addition of 5 μM AM 251 (**blue**). **(D)** Group data showing activation of CB1 receptor by Win 55,212-2 decreases IPSC peak amplitude and the blocking of CB1 receptor by AM 251 reverses Win 55,212-2-induced inhibition and causes a small increase of IPSC amplitude from ACSF (*n* = 6). **(E)** Percent change of IPSC peak amplitude in the presence of 5 μM Win 55,212-2 and with the further addition of 5 μM AM 251 (*n* = 6). **(F)** Representative traces of IPSC in control (**black**) and in the presence of 5 μM AM 251 (**magenta**) and additionally 5 μM Win 55,212-2 (**violet**). **(G)** Group data showing AM 251 blockade of tonic CB1 receptor activation increases IPSC peak amplitude and further addition of Win 55,212-2 has no effect (*n* = 7). **(H)** Percent change of IPSC peak amplitude in the presence of 5 μM AM 251 and the further addition of 5 μM Win 55,212-2 (*n* = 7). **(I)** Modulation of CB1 activity does not change evoked IPSC latency, jitter, half-width, rise tau or decay tau. *N* = 13 for ACSF, six for Win 55,212-2 and 7 for AM 251. On all graphs, **P < 0.01 and *P < 0.05 *vs* ACSF.

Endocannabinoid activation robustly suppressed IPSC amplitude and we determined the dose response curves for Win 55,212-2 inhibition of HDB-granule cell IPSCs across the concentration range of 0.1 – 15 μM. The presence of Win 55,212-2 dose dependently reduced the magnitude of optically evoked IPSC (*F*_6_,_27_ = 23.15, *P* < 0.01, *n* = 4 for 0.1 μM and *n* = 5 for other concentrations, one-way ANOVA; Figure 5A,B) with threshold dose of 0.5 μM (*n* = 5) and maximal inhibition obtained with 10 μM Win 55,212-2. The highest concentration of Win 55,212-2 inhibiting the evoked IPSCs by 57.7 ± 6.9% (15 μM; *n* = 5; Figure 5A,B). The concentration of Win 55,212-2 that gave half maximal effective concentration (EC50) was ∼2.5 μM (Figure 5B). To examine if selective CB1 receptor antagonist AM 251 can block Win 55,212-2-induced inhibition of IPSC, granule cells treated with 5 μM Win 55,212-2 were further treated with AM 251 (5 μM). The addition of AM 251 abolished Win 55,212-2-induced inhibition of IPSC and induced a 9.2 ± 1.5% increase of peak amplitude from ACSF (*n* = 6, *P* < 0.01; Figures 5C–E). This suggests HDB to granule cell GABAergic synapse are under tonic endocannabinoid modulation. We further addressed this by testing the effect of 5 μM AM 251 alone on the peak amplitude of optically evoked IPSCs. After bath application of the antagonist, we recorded a 10.8 ± 1.3% IPSC enhancement in the presence of AM 251 (*n* = 7, *P* < 0.01; Figures 5F–H). The further addition of Win 55,212-2 did not cause any effect, indicating that Win 55,212-2-induced inhibition was mediated by CB1 receptor activation.

The IPSC kinetics of latency, jitter, half-width of peak amplitude, rise tau and decay tau were not statistically different between ACSF, 5 μM Win 55,212-2 and 5 μM AM 251, respectively (ANOVA, *P* > 0.05, *n* = 13 for ACSF, *n* = 6 for Win 55212-2 and *n* = 7 for AM 251, Figure 5I). Coefficient of variation (CV), standard deviation/mean of IPSC amplitude from the averaged 20 trials for individual cell was 0.095 ± 0.009 for ACSF, and increased to 0.153 ± 0.017 for Win 55212-2 (5 μM, *n* = 6, *P* < 0.01, paired *t*-test). The increased CV (accompanied by an increased PPR by Win 55212-2 described below) and absence of any change in IPSC kinetics suggests a primarily presynaptic modulatory effect of CB1 receptor. Together, these results indicate that the endocannabinoid receptor activation can potently inhibit GABAergic cortical input to the bulb and the synapse is under a modest endogenous tonic cannabinoid activation.

### Endocannabinoid Receptor Modulates HDB-Granule Cell Short-Term Plasticity

**FIGURE 6:**
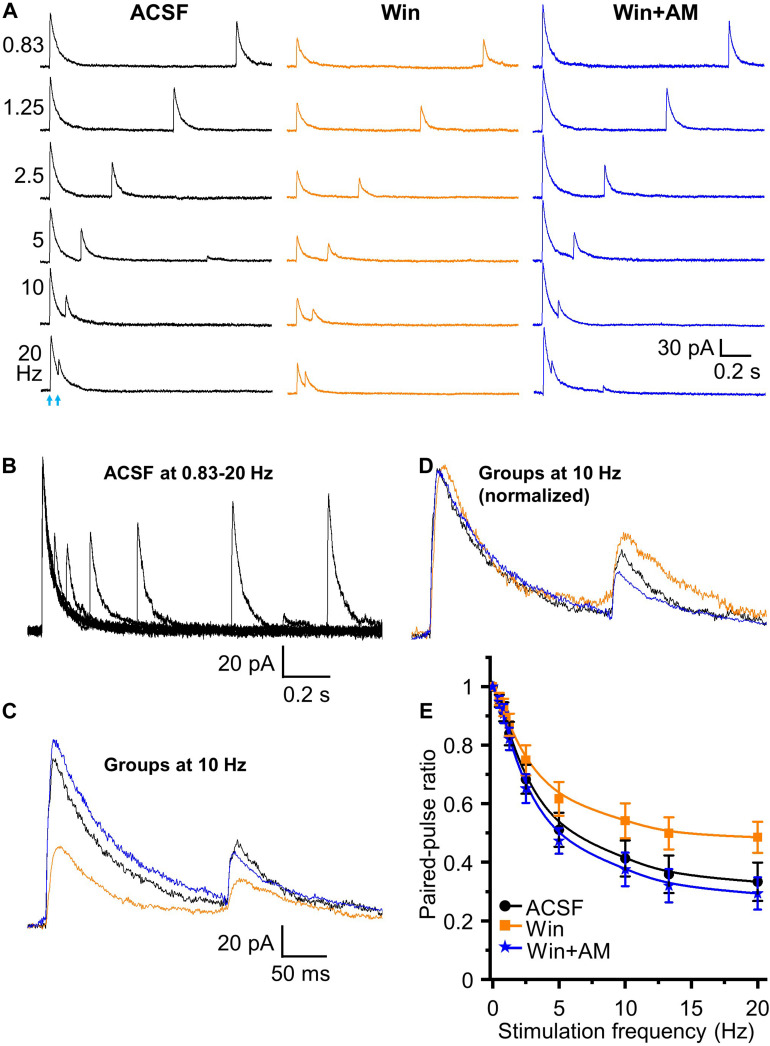
Endocannabinoids modulate short term plasticity of HDB GABAergic inputs to granule cells. **(A)** Representative traces of initiated IPSCs by paired-pulse optical stimulation at frequencies of 0.83 to 20 Hz in ASCF, Win (Win 55,212-2, 5 μM) and Win + AM (AM 251, 5 μM). **(B)** Superimposed traces in ACSF at different frequencies showing short-term depression in the peak amplitude of test IPSCs with increase of frequencies from 0.83 to 20 Hz in ACSF. **(C)** Superimposed representative traces in ACSF (**black**), Win (**orange**) and Win + AM (**blue**) at 10 Hz. **(D)** Representative traces of control IPSC in Win and Win + AM at 10 Hz normalized to ACSF showing relative changes in test (second) stimulus IPSC peak amplitudes. **(E)** Group data showing PPR decreases with increase of stimulation frequencies from 0 to 20 Hz (0, 0.5, 0.83, 1.25, 2.5, 5, 10, 13.3, 20 Hz) in ACSF, Win and Win + AM. Activation of CB1 receptor increases PPR. Blocking of CB1 receptor reversed Win 55,212-2-induced increase of PPR and caused a further decrease of PPR from ACSF. *N* = 6.

**FIGURE 7:**
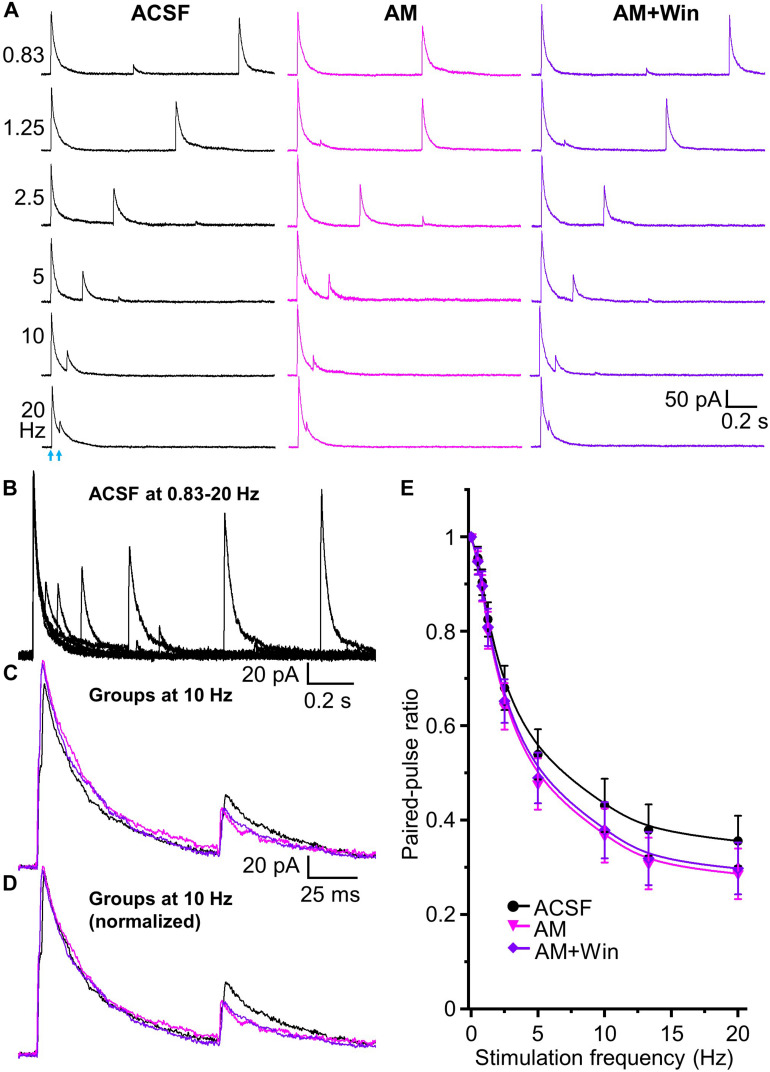
Tonic endocannabinoid modulation at the HDB-granule cell synapses. **(A)** Representative traces of evoked IPSCs by paired-pulse optical stimulation at frequencies of 0.83 to 20 Hz (corresponding to interstimulus intervals of 1.2 to 0.05 s) in ASCF, AM (AM 251, 5 μM) and AM + Win (Win, Win 55,212-2, 5 μM). **(B)** Superimposed traces in ACSF at different frequencies showing short term depression in the peak amplitude of test IPSCs with increase of frequencies from 0.83 to 20 Hz. **(C)** Superimposed representative traces in ACSF (**black**), AM (**magenta**) and AM + Win (**blue**) at 10 Hz are superimposed to show slightly different control and test IPSCs. **(D)** Representative traces of control IPSC in AM and AM + Win at 10 Hz normalized to ACSF showing relative changes in test (second) stimulus IPSC peak amplitudes. **(E)** Group data showing PPR decreases with increase of stimulation frequencies from 0.5 to 20 Hz (0, 0.5, 0.83, 1.25, 2.5, 5, 10, 13.3 and 20 Hz) or intervals of 2 to 0.05 s in ACSF, AM and AM + Win. AM 251 to block CB1 receptor decreases PPR from ACSF. Win 55,212-2 does not cause any further change of PPR in the presence of AM 251. *N* = 7.

Does the endocannabinoid suppression of GABA release modulate the magnitude of short-term depression at the HDB-granule cell synapse? To investigate this, we first used the CB1 receptor agonist Win 55,212-2. A concentration of 5 μM Win 55,212-2 was used for this experiment as this result in a robust, but sub-maximal, CB1 receptor activation. At this concentration, IPSC amplitude is reduced to 43.7 ± 4.2% of the baseline level (approximately EC70 for Win 55,212-2 at this synapse; Figures 5A,B). When paired-pulse optical stimuli were applied, bath application of 5 μM WIN 55,212-2 significantly attenuated short-term depression at all frequencies above 2.5 Hz (i.e., increase in the PPR; *F*_1_,_90_ = 63.05, *P* < 0.01, *n* = 6; two-way-ANOVA, Win 55,212-2 *vs* ACSF at different frequencies; Figure 6). Granule cells further treated with the CB1 receptor antagonist AM 251 (5 μM) abolished Win 55,212-2-induced inhibition of IPSC. When AM 251 is present short-term depression was slightly enhanced (i.e., decrease in the PPR; *F*_1_,_90_ = 125.18, *P* < 0.01, *n* = 6; two-way-ANOVA, Win + AM *vs* Win; *F*_1_,_90_ = 7.22, *P* < 0.01 (0.00858), *n* = 6; two-way-ANOVA, Win + AM *vs* ACSF; Figure 6) further supporting the observation that the HDB to granule cell GABAergic synapse is under modest endogenous tonic cannabinoid action. The PPR steadily decreased with increased stimulation frequency in ACSF, Win and Win + AM (*F*_8_,_45_ = 184.66, 127.74, and 260.19, respectively, *P* < 0.01, one-way ANOVA, Figure 6E). To validate this, the drugs were also applied in the alternative order (antagonist then agonist). The antagonist AM 251 (5 μM) modestly decreased PPR (*F*_1_,_108_ = 25.29, *P* < 0.01, *n* = 7, two-way-ANOVA, AM 251 *vs* ACSF; Figure 7). After CB1 receptor was blocked by AM 251, bath applied Win 55,212-2 for 15 – 25 min did not induce any further change in the IPSC (*F*_1_,_108_ = 0.99, *P* > 0.05, *n* = 7; two-way-ANOVA, AM 251 *vs* AM + Win; *P* > 0.05). The PPR steadily decreased with increased stimulation frequency in ACSF, AM and AM + Win (F_8_,_54_ = 248.91, 292.54, and 274.18, respectively, *P* < 0.01, one-way ANOVA, Figure 7E).

Those results show that activation of CB1 receptor by exogenous application of Win 55,212-2 significantly decreases HDB-granule cell IPSC peak amplitude and increases PPR (decreases short-term depression). Deactivation of CB1 receptor by AM 251 (in ACSF or after pretreatment of Win 55,212-2) increases IPSC amplitude and decreases PPR (increases short-term depression). This demonstrates robust bidirectional control of frequency dependent STP by endocannabinoids and indicates the synapse is under tonic endogenous CB1 receptor activation.

## Discussion

The inhibitory granule cells of the olfactory bulb are regulated by dendrodendritic reciprocal synapses with output neurons of the bulb, mitral/tufted cells. However, they are also the target of massive feedback projections from primary olfactory cortex (Boyd et al., 2012; Ennis et al., 2015). These feedback projections consist of modulatory inputs (serotonergic, noradrenergic, and cholinergic) as well as heavy glutamatergic projections from anterior olfactory nucleus, piriform cortex, and entorhinal cortex amongst other regions. However, there is a recent appreciation that granule cells also receive abundant long-range GABAergic feedback projections from the HDB (Ma and Luo, 2012; Nunez-Parra et al., 2013). In this study, we show that the HDB to granule cell synapse undergoes significant frequency dependent short-term depression and that both inhibitory currents and STP are modulated by the endocannabinoid system.

### HDB GABAergic Inhibition of Granule Cells in Olfactory Bulb

The HDB sends abundant inhibitory GABAergic projects to granule cells (Ma and Luo, 2012; Nunez-Parra et al., 2013) and disruption of this centrifugal inhibition from HDB impairs olfactory discrimination (Nunez-Parra et al., 2013). Optical activation of HDB GABAergic inhibitory fibers has been reported to initiate IPSC in granule cells (Nunez-Parra et al., 2013; Sanz Diez et al., 2019), as well as other olfactory bulbar neurons: deep short axon cells and periglomerular cells (Sanz Diez et al., 2019). Since granule cells inhibit mitral/tufted cells, HDB inhibition to granule cell could cause mitral/tufted cells disinhibition, potentially allowing enhanced M/TC excitability. Indeed, studies suggest that HDB electric stimulation excites mitral cells in olfactory bulb (Kunze et al., 1991; Zhan et al., 2013). However, electric stimulation could activate different projections including inhibitory (such as GABAergic) and excitatory projections (cholinergic) from HDB and other adjacent regions.

Since MCs project to higher processing areas in the cortex (piriform and other olfactory cortical domains), selective optogenetic stimulation of inhibitory GABAergic projections to granule cells could result in elevated excitability of mitral cells through the disinhibition of GC inhibitory action on the mitral cell. Since CB1 receptor activation inhibits optical stimulation-induced HDB inhibition to granule cells, that would reduce inhibition of GCs by the cortex and thus reduce the magnitude of potential granule cell disinhibition of mitral cells. However, the circuits between HDB, granule cells and mitral cells may be much more complicated that this simple prediction makes. Optical stimulation could activate HDB projections directly to mitral cells that inhibit mitral cells simultaneously with inhibition of granule cells (and other interneurons) in the bulb. CB1 receptors could be present on multiple of these types of cells and act directly or indirectly. Indeed, a recent study has reported that CB1 receptor activation strongly modulate mitral cell activity (Wang et al., 2019). Thus, while the simple prediction is that endocannabinoids will act overall to reduce mitral cell excitability the experiments need to be performed carefully due to confounding alternative network effects.

Horizontal limb of the diagonal band of Broca GABAergic and cholinergic neurons project to olfactory bulb (Linster and Hasselmo, 2000; Ma and Luo, 2012; Nunez-Parra et al., 2013; Rothermel et al., 2014; Case et al., 2017; Sanz Diez et al., 2019). Cholinergic neurons predominantly project to the glomerular and internal plexiform layer of olfactory bulb, with sparse projections in GCL (Case et al., 2017). ACh and GABA are expressed and co-released from synapses of forebrain (HDB) cholinergic neurons to cortical layer 1 interneurons (Saunders et al., 2015). Co-expression of GABA and ACh from HDB cholinergic neurons to olfactory bulb axon terminals has also been reported to act on deep short axon cells in the olfactory bulb (Case et al., 2017). However, when we recorded postsynaptic currents in granule cell in GCL at multiple holding potentials to map optically evoked inward and outward current from selective ChR2 expression in HDB GABAergic neurons, the observed currents were not significantly affected by cholinergic or glutamatergic receptors antagonists. However, evoked currents were completely blocked by a GABA_*A*_ receptor antagonist, suggesting that HDB-granule cell synapses may release a single transmitter of GABA in contrast to targeting of the deep short axon cells (Case et al., 2017). We cannot exclude the possibility that granule cells are innervated by a large number of GABAergic only synapses commingled with a small number of HDB synapses that do co-release GABA and ACh. However, if there is a mix of GABA only versus co-release terminals, it would have to be a very low level of dual GABA/ACh release to have been undetectable using our patch clamp techniques. More likely is that GABA only and GABA/ACh may target different cell populations with GABA only primarily on granule cells and GABA/ACh acting on deep short axon cells. Basal forebrain (and other brain regions) cholinergic axon terminals had often been reported to exhibit co-transmission of ACh and GABA (Saunders et al., 2015; Case et al., 2017; Takács et al., 2018). While there is robust release of GABA, release of ACh is more sparse and selective to specific neuronal targets, e.g., 3/11 of deep short axon cells (Granger et al., 2018; Sanz Diez et al., 2019). Therefore, we could miss ACh co-transmission with GABA from HDB GABAergic axon terminals if there is a heterogeneity in granule cells with some receiving ACh and others GABA only due to the sample sizes in the study. More sensitive broad “mass action” methods, such as calcium imaging where hundreds of cells can be imaged, will be needed to parse out the possibility of subpopulation heterogeneity.

### Short-Term Depression of HDB-Granule Cell Synapse

Short-term plasticity plays an important role in neural functions, including sensory processing (Zucker and Regehr, 2002; Abbott and Regehr, 2004; Wilson et al., 2004; Li et al., 2018). Electrical stimulation of centrifugal axons projecting into the bulb induces long-term (Gao and Strowbridge, 2009; Cauthron and Stripling, 2014) and STP (Balu et al., 2007) of granule cell synaptic responses. Electrical stimulation activates all input fibers such that the plasticity of specific cortical synapses cannot be deduced as these inputs comprise a mix of glutamatergic, cholinergic, GABAergic, noradrenergic and serotonergic inputs (Fallon and Moore, 1978; Macrides et al., 1981; McLean et al., 1989; Ennis et al., 2015).

We recently showed optical stimulation of excitatory centrifugal axons from olfactory cortex to olfactory bulbar granule cell robustly induces short-term facilitation (Zhou et al., 2018). The present study used gene-targeted viruses to drive ChR2 expression selectively in GABAergic HDB-granule cell axons to investigate plasticity at the GABAergic synapse. In contrast to the glutamatergic cortex to granule cell synapse which exhibited short-term facilitation, the GABAergic HDB to granule cell synapse shows robust short-term depression. Functionally, this may result in excitatory feedback being amplified across sniff cycles while inhibitory feedback is reduced.

Investigation of STP using optical stimulation of ChR2 positive axons to evoke IPSCs has frequency limitations of ChR2 photoactivation due to the kinetics of the ChR2 channel itself: ChR2 activation (∼2 ms) and inactivation (∼10 ms) (Gunaydin et al., 2010). The strength of ChR2 stimulation could decline at high frequencies due to incomplete repolarization of the ChR2 membrane potential (Grossman et al., 2011). This limitation in our study is circumvented by using short (2 ms) light stimuli as suggested by Malyshev et al. (2015), and awareness of the optical stimulation of ChR2 frequency limit of up to 50 Hz (Jackman et al., 2014) or as high as ∼70–100 Hz in some studies (Malyshev et al., 2015). Our experience is that rundown starts to become detectable at 40–50 Hz and limited our study to 20 Hz or less.

The reduction in the magnitude of consecutive IPSCs could represent presynaptic depression, or desensitization of postsynaptic receptors or rundown artifacts at high frequency (Liewald et al., 2008). Previous research has also implicated AAV package serotype in the rundown of GABAergic Purkinje cells. The authors observed that optical artifactual synaptic depression is eliminated in synapses from GABAergic Purkinje cells to deep cerebellar nuclei neurons were similar when ChR2 was expressed with AAV9, instead of other AAV expression vectors (Jackman et al., 2014). Thus, the present study used the AAV9 exclusively. Additionally, in our previous study with the same methodology on glutamatergic synapses we observed paired pulse facilitation of olfactory cortex-granule cell synapse by optical stimulation across the same frequency range (Zhou et al., 2018). Therefore, if any artifactual rundown of the responses in this study with increasing frequency would have to be specific to GABAergic synapses, and also modulated by CB1 receptors as those influence the magnitude of short-term depression. Thus, while we cannot exclude completely the possibility of amplitude decline during repeated flash ChR2 stimulation at high frequencies in the present study as an artifact of ChR1 or the AAV serotype, we consider this to be highly unlikely.

### CB1 Receptor Activation Modulates Short-Term Plasticity of HDB-Granule Cell Synapse

The observation of excitatory and inhibitory short-term potentiation accruing in opposite directions means that any modulatory action that occurs across short-term potentiation could reduce or enhance this bidirectional regulation. Endocannabinoids have been shown to potently regulate synaptic transmission. In other brain regions, CB1 receptor activation inhibits synaptic transmission by reducing neurotransmitter release (Auclair et al., 2000; Azad et al., 2003). Consistent with this observation, we observed a strong endocannabinoid inhibition of GABAergic transmission at the HDB projections onto granule cells.

Cannabinoid type 1 receptor is heavily expressed in olfactory bulb, particularly in GCL (Freundt-Revilla et al., 2017; Wang et al., 2019), while CB2 is not expressed within the bulb (Gong et al., 2006). Indeed, the intrabulbar control of inhibitory inputs to mitral cells in glomeruli is strongly modulated by CB1 receptor activity (Wang et al., 2012, 2019). The non-selective agonist (and selective antagonist) used in this study are likely operating through CB1 receptors as this is the dominant type in the bulb.

Short-term plasticity is strongly determined by neurotransmitter release probability in the presynaptic terminals, with high release probability producing short-term depression and low release probability producing short-term facilitation (Stevens and Wang, 1995; Debanne et al., 1996; Kim and Alger, 2001). PPR is a well-characterized measure of the probability of neurotransmitter release and fairly specific for presynaptic mechanisms (Debanne et al., 1996; Kim and Alger, 2001). Decreasing the probability of release should cause a consistent increase in PPR and vice versa. This synaptic vesicle depletion model accounts for the basic properties of paired-pulse depression observed at many synapses. The more vesicles fuse in response to the initial control stimulus, the more depletion of the readily releasable pool and the fewer vesicles released by the test stimulus, leading to much smaller peak amplitude to test pulse than to the control, and the more pronounced the paired-pulse depression. In the present study, the inhibition of IPSCs by CB1 receptor activation is consistently accompanied by an increase in the PPR, which we interpret as a reduction in the probability of neurotransmitter release by endocannabinoids.

Cannabinoid type 1 receptor activation has been known to reduce readily releasable pool (García-Morales et al., 2015), which could contribute to the decreased paired-pulse depression via a reduction in available transmitter vesicles during the first stimulus. CB1 receptor activation is also reported to reduce inhibition of GABA uptake and so potentiating GABAergic transmission leading to an increased paired-pulse depression (Al-Hayani and Davies, 2002). However, this is opposite to our observations on endocannabinoid activation on paired-pulse depression in HDB-granule cell synapse. This suggests that the role of CB1 on GABA uptake may be minimally, or not, involved in the reduced paired-pulse depression in this study. Other presynaptic mechanisms including inactivation of release sites, inhibition or inactivation of calcium channels and/or postsynaptic mechanisms such as receptor saturation and desensitization could contribute to the CB1 modulation of short-term depression. The detailed mechanistic targets of CB1 at this synapse remain to be investigated.

Endocannabinoids in the olfactory bulb are likely to be synthesized and released from postsynaptic neurons as a result of cellular excitation. Different types of neurons in the olfactory bulb including external tufted cells in glomerular cell layer (Heinbockel et al., 2016), granule cells, deep short axon cells (Pouille and Schoppa, 2018), and mitral cells (Heinbockel and Wang, 2015; Freundt-Revilla et al., 2017) could be potential endocannabinoid sources in the olfactory bulb. Mitral cells show cluster firing riding on a slow long-lasting depolarization and that excite granule cells and deep short axon cells (Carlson et al., 2000; Schoppa and Westbrook, 2001). The repetitive activity of mitral/tufted cells acting on granule cells could induce the tonic release of endocannabinoids, leading to tonic inhibition of IPSCs by activation of CB1 receptor of HDB terminals. Alternatively, excitatory glutamatergic cortical input which undergoes short-term Cav2.1 dependent potentiation (Zhou et al., 2018) may depolarize granule cells and induce endocannabinoid release to dampen HDB inhibition to the granule cells.

### Functional Implication of HDB-Granule Cell Synapse Modulation

Granule cell activation is strongly dependent on centrifugal input and granule cells may be critically involved in mediating centrifugal modulation of sensory processing in the main olfactory bulb (Burton, 2017). Granule cells are situated to respond dynamically to multiple sources of synaptic input to shape olfactory bulb output to HDB and other brain regions across sniff cycles due to excitatory feedback potentiation (Zhou et al., 2018) and the inhibitory short-term depression observed here. Frequency-dependent STP of HDB→granule cell synapse as shown in this study may determine a balance of excitation and inhibition of granule cells based on behavioral state. Animals adjust their sniffing according to the salience of odorant stimuli, engaging in high frequency bouts in response to novel stimuli (Verhagen et al., 2007). As sniff rate increases, the HDB→granule cell synapse could be progressively depressed reducing the disinhibition of olfactory bulb output neurons. Enhancement of the competing olfactory cortex-granule cell excitatory synapses (Zhou et al., 2018) might in contrast work to enhance granule cell excitation and feed-forward inhibition of M/TC firing by granule cells. The observation of STP in both excitatory and inhibitory cortical feedback coupled with modulation by endocannabinoids and other modulatory transmitters suggests control of granule cell function is exquisitely balanced by competing inputs. Behavioral states may modulate the balance of these factors to tilt the olfactory bulb signal-to-noise ratios to facilitate odor detection, processing and formation of sensory memory.

## Data Availability Statement

The raw data supporting the conclusions of this article will be made available by the authors, without undue reservation.

## Ethics Statement

The animal study was reviewed and approved by the University of Maryland Institutional Animal Care and Use Committee.

## Author Contributions

FZ and AP designed and analyzed the experiments and wrote the article. FZ performed the experiments. Both authors contributed to the article and approved the submitted version.

## Conflict of Interest

The authors declare that the research was conducted in the absence of any commercial or financial relationships that could be construed as a potential conflict of interest.
